# Supplementary material for: A bio-inspired visuotactile neuron for multisensory integration
Source: Nat Commun. 2023 Sep 15;14:5729. doi: 10.1038/s41467-023-40686-z (PMC10504285; doi:10.1038/s41467-023-40686-z)
Supplement: Supplementary file 1 — Supplementary Information [file 41467_2023_40686_MOESM1_ESM.pdf]

Supplementary Information

A Bio-inspired Visuotactile Neuron for Multisensory Integration

Muhtasim Ul Karim Sadaf[1,#], Najam U Sakib[1,#], Andrew Pannone[1], Harikrishnan Ravichandran[1], and Saptarshi Das[1,2,3,4,]*

[1]Engineering Science and Mechanics, Penn State University, University Park, PA 16802, USA

[2]Electrical Engineering, Penn State University, University Park, PA 16802, USA

[3]Materials Science and Engineering, Penn State University, University Park, PA 16802, USA

[4]Materials Research Institute, Penn State University, University Park, PA 16802, USA

[#] Equal Contribution

[*] Corresponding author: sud70@psu.edu

Supplementary Information 1

Supplementary Table 1: Demonstration of Multisensory Integration

No.	Materials and Devices Used		Demonstration of Key Multisensory Integration Features			Spike Encoding	Ref.
	Sensory input 1	*Sensory input 2*	Super-additivity	Inverse Effectiveness Effect	Temporal Congruency		
1.	MoS_2 FET	Piezoresistor	✓	×	×	×	[1]
	Visual	*Tactile*					
2.	MoS_2 FET	Commercial motion sensor	×	✓	×	×	[2]
	Visual	*Vestibular*					
3.	SONOS FinFET with Si_3N_4 charge trap layer		×	×	×	×	[3]
	Visual	*Thermal*					
4.	MoS_2 FET	PTFE/Cu triboelectric nanogenerator	×	×	×	×	[4]
	Visual	*Tactile*					
5.	$CsPbBr_3/TiO_2$ floating gate FET		×	×	×	×	[5]
	Visual	*Thermal*					
6.	Self-powered perovskite (PSK) on ITO with SnO_2, Spiro-OMeTAD, and Au layers		×	×	×	✓	[6]
	Visual	*Tactile*					
7.	P3HT/PEO NW FET, PVDF-HFP, EMIM-TFSI ion gel		×	×	×	×	[7]
	Visual	*Thermal*					
8.	Camera	SWCNT strain sensor	×	×	×	×	[8]
	Visual	*Tactile*					
**9.**	MOCVD MoS_2 FET	Kapton/Al-based tactile sensor	✓	✓	✓	✓	This Work
	Visual	*Tactile*					

Supplementary Figure 1

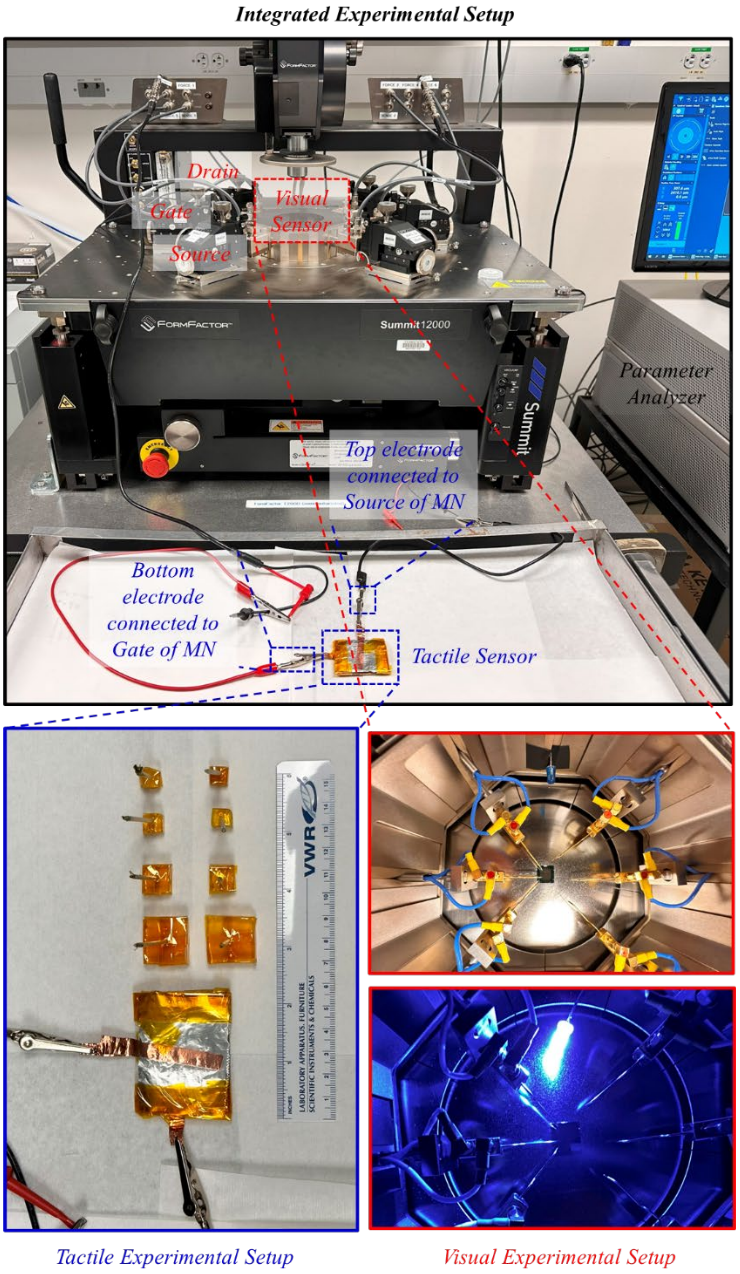

Supplementary Figure 1. Experimental setup for the demonstration of visuotactile multisensory integration. Our tactile sensor is comprised of a stack of commercially available Kapton, and aluminum foil separated by an air gap; PDMS stamps with different surface areas were prepared to serve as the touch stimuli. Monolayer MoS_2 memtransistors serve as the optical sensor and are also used to construct the visuotactile spike encoding circuit. The MoS_2 memtransistors, fabricated on a 1 cm × 1 cm substrate, are placed inside the Form Factor probe station, and are connected *via* probe tips/arms to external SMUs. Similarly, the top and bottom contacts of the tactile sensor are connected to SMUs via alligator clips. Finally, appropriate adapters are used to connect/short the relevant SMUs. A Keysight B1500 parameter analyzer is used for sourcing and measuring current/voltage trough the respective SMUs.

Supplementary Figure 2

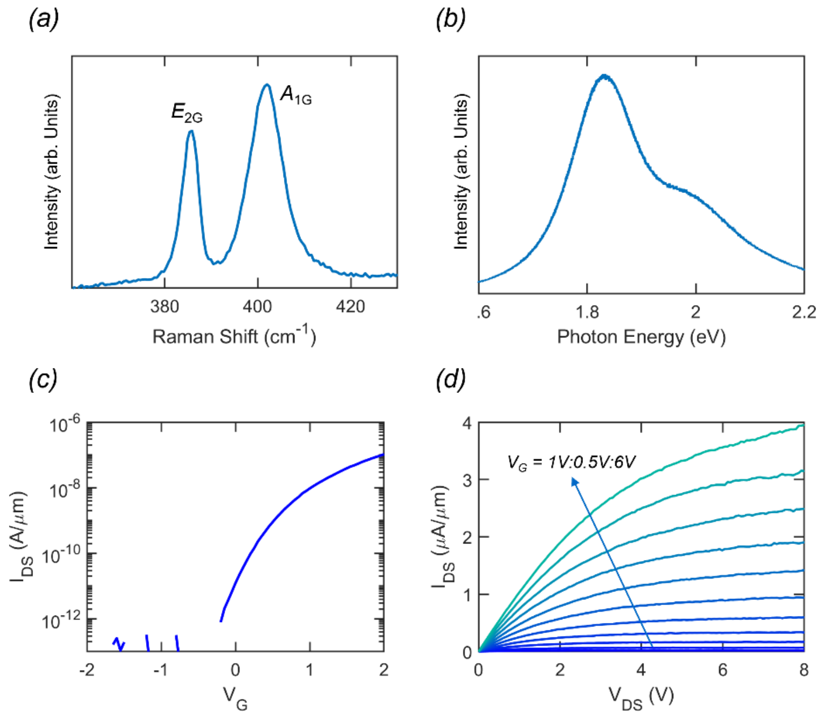

Supplementary Figure 2. Optical and electrical characterization of the monolayer MoS_2 photo-memtransistor. a) Raman spectra of the monolayer MoS_2, obtained using a 532 nm laser, shows the in-plane E_{2G} and out-of-plane A_{1G} peaks at 384.24 and 401.53 cm^{-1}, respectively, with a peak separation of ~17 cm^{-1}, which corresponds to monolayer MoS_2 film. b) Photoluminescence (PL) spectra for monolayer MoS_2 with a peak at 1.83 eV, which also confirms having a monolayer film. c) Transfer characteristics i.e., drain current (I_{DS}) versus gate voltage (V_G), of the monolayer MoS_2 photo-memtransistor in dark condition for a channel length of 1 µm and width of 5 µm. Source-to-drain voltage (V_{DS}) was 1 V. Key device parameters like threshold voltage (V_{TH}), peak field-effect electron mobility (μ_N), and subthreshold swing (SS) were measured. We obtained a V_{TH} of ~0.8 V using the iso-current method at 10 nA/µm, an SS value of ~252 mV/dec for two orders of magnitude change in I_{DS}, and a μ_N of ~7.78 cm^2/Vs was obtained from the peak transconductance method. d) Output characteristics, i.e., I_{DS} vs V_{DS}, with V_G ranging from 1 V to 6 V in 0.5 V increments.

Supplementary Figure 3

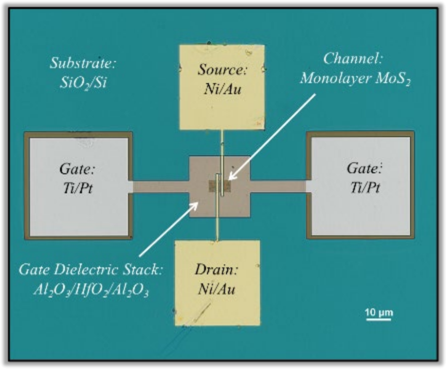

Supplementary Figure 3. MoS_2 photo-memtransistor. Plane-view optical micrograph of a monolayer MoS_2-based photo-memtransistor showing the MoS_2 channel and the floating-gate stack consisting of Al_2O_3/HfO_2/Al_2O_3 on a Ti/Pt back-gate electrode.

Supplementary Figure 4

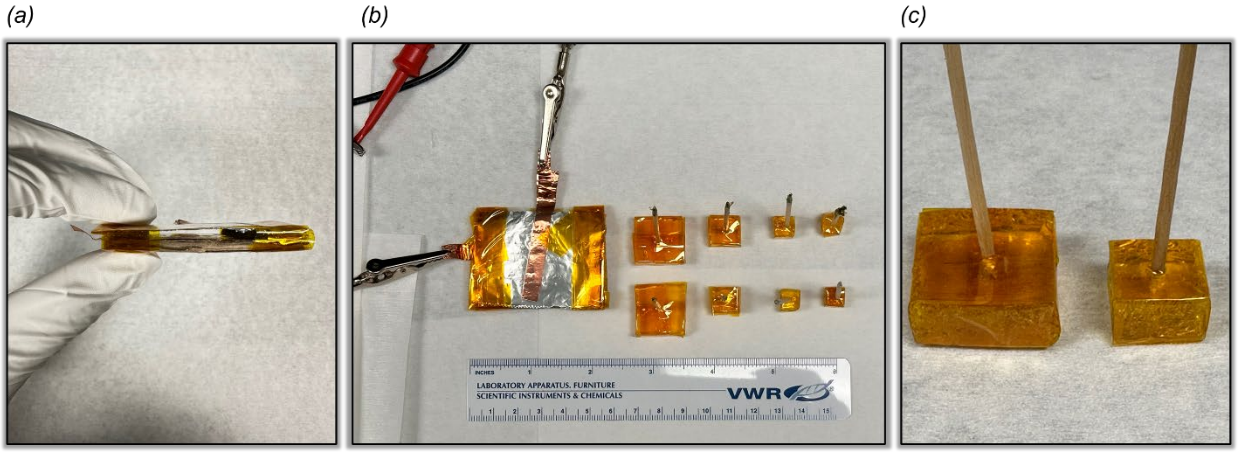

Supplementary Figure 4. Triboelectric tactile sensor and touch stimuli. a) Side view of the tactile sensor, with Kapton on the bottom layer on top of a supporting substrate with spacers separating it from the top aluminum layer sitting below a separate supporting substrate. b) Top view of the tactile sensor with copper extensions from the top and bottom layer and PDMS stamps used for the experiment representing different touch stimuli. c) Closer image of PDMS stamps.

Supplementary Figure 5

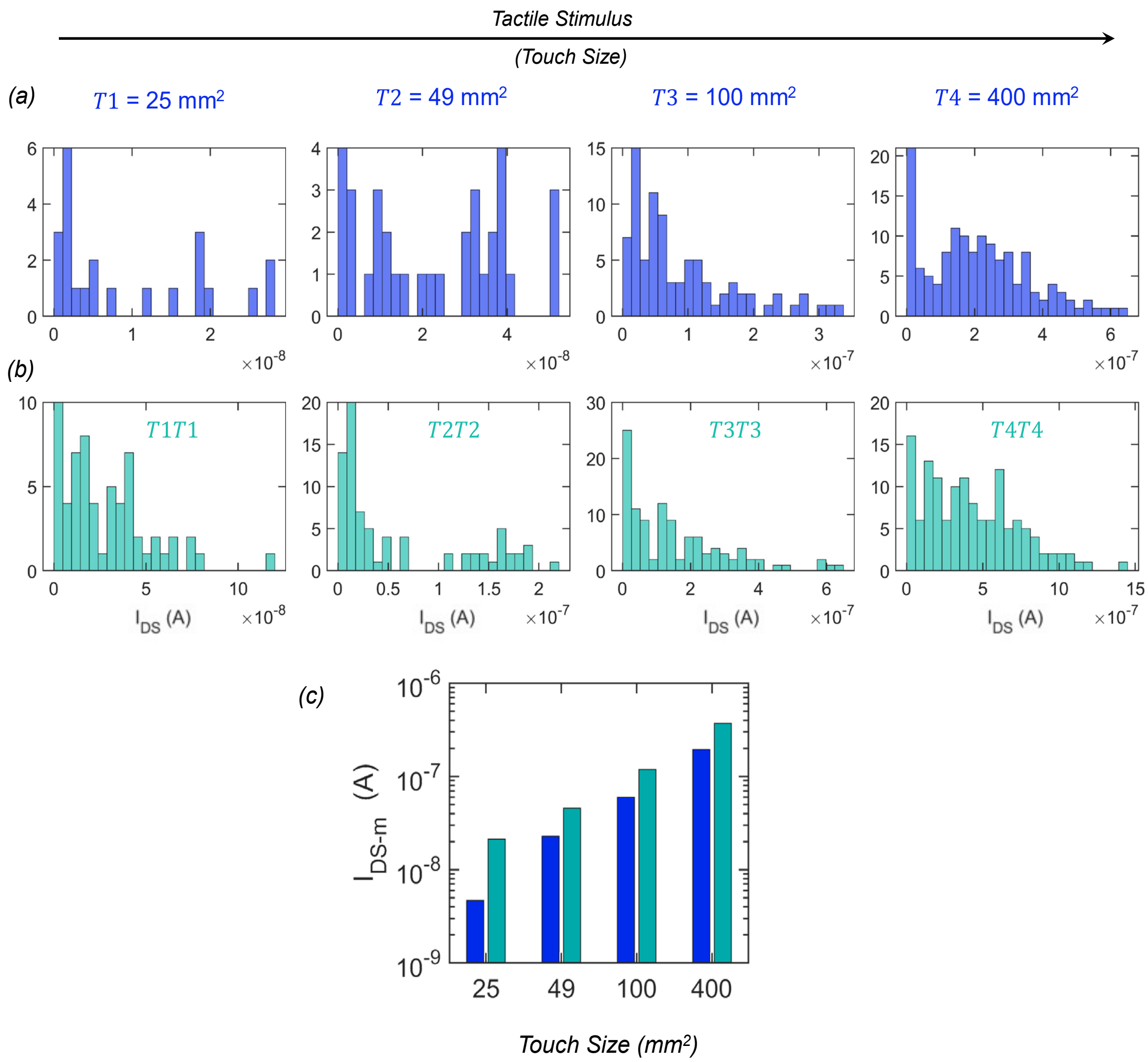

Supplementary Figure 5. Tactile response of MN in dark. Histogram of I_{DS} spikes for a) single touch and b) dual touch inputs with different tactile stimuli, $T1 = 25$ mm^2, $T2 = 49$ mm^2, $T3 = 100$ mm^2, and $T4 = 400$ mm^2, respectively, in dark. c) Bar plot of median spike magnitude ($I_{\mathrm{DS-m}}$) as a function of the strength of the tactile stimuli with the left bar in each group showing response for single touch and the right bar indicating the response for the dual touch with different tactile stimuli (T) in dark.

Supplementary Figure 6

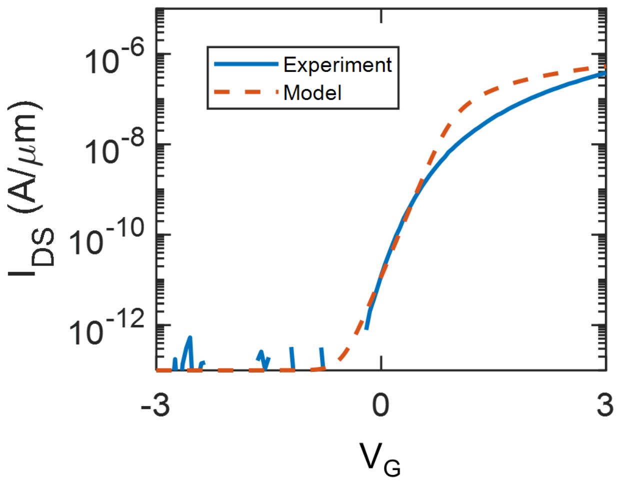

Supplementary Figure 6. The virtual source (VS) model. VS model fitting of the experimental transfer characteristics for monolayer MoS_2 photo-memtransistor.

Supplementary Figure 7

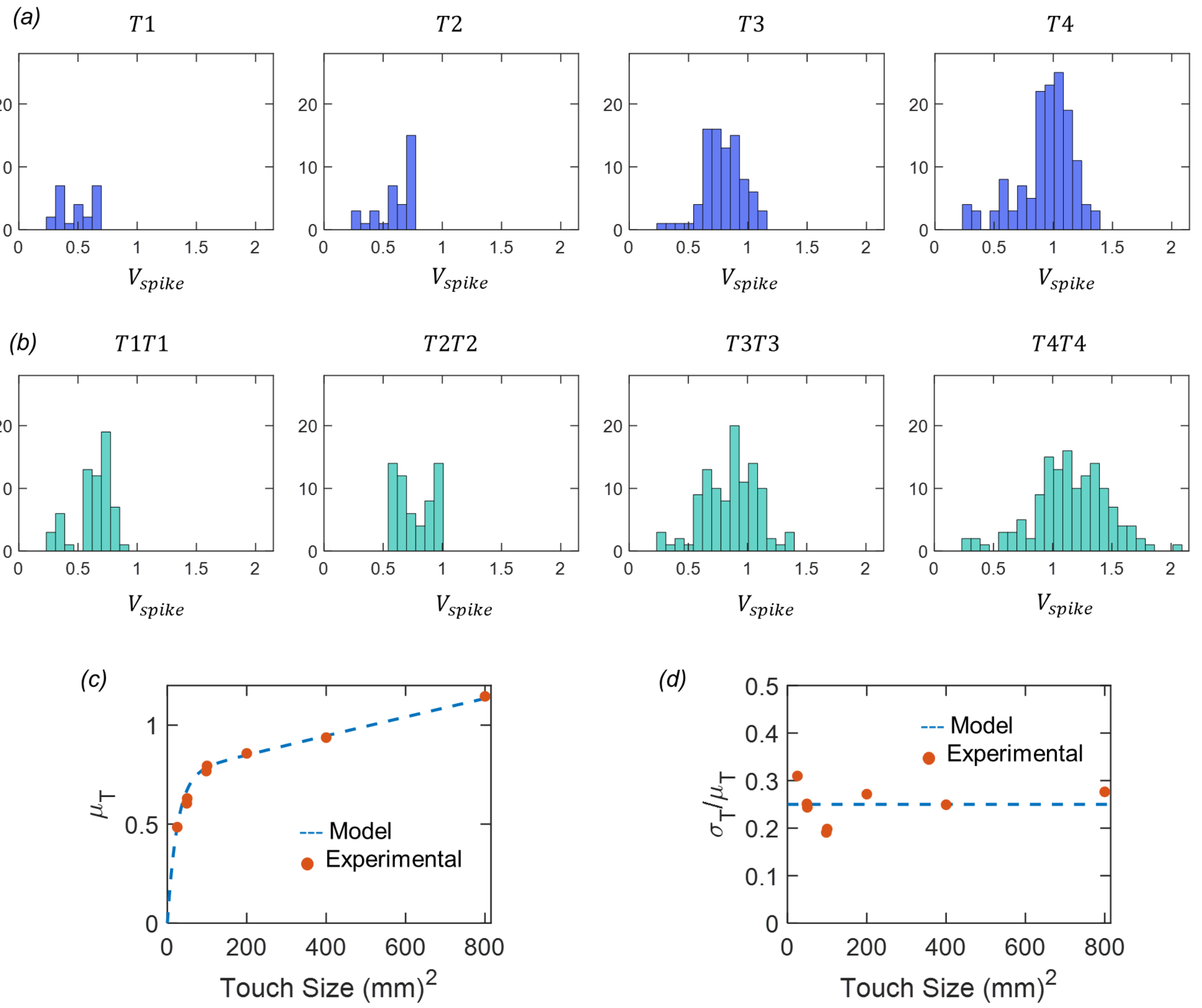

Supplementary Figure 7. Modeling of triboelectric tactile response. Histograms of V_{spike} extracted from the I_{DS} spikes using the VS model for a) single (T) and b) dual (TT) touches of various strengths. The distributions of V_{spike} were described using Gaussian functions with μ_{T} and σ_{T} as the mean and standard deviation, respectively. c) μ_{T} and d) $\sigma_{\mathrm{T}}/\mu_{\mathrm{T}}$ as a function of T along with empirical model fitting.

Supplementary Figure 8

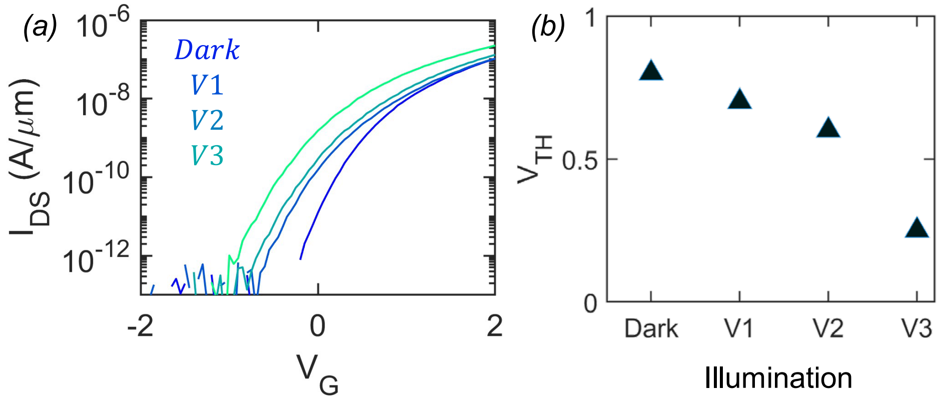

Supplementary Figure 8. Photoresponse from monolayer MoS_2 photo-memtransistor. a) Transfer characteristics of a MoS_2 photo-memtransistor measured pre-illumination and after exposure to various visual cues. Visual cues were generated from a light emitting diode (LED) by feeding an input current of 100 mA for $V1$ = 1 s, $V2$ = 2 s, and $V3$ = 10 s. Note that the shifts in the transfer characteristics are due to trapping of photo-generated carriers at the MoS_2/dielectric interface, which is known as the photo-gating effect and is responsible for visual memory offered by MoS_2 photo-memtransistors. b) Extracted threshold voltage (V_{TH}) at an iso-current of 10 nA from the transfer characteristics in (a) at different visual stimuli starting from dark; a decrease in V_{TH} with longer illumination is shown.

Supplementary Information 2

The photogating effect is a direct consequence of gate-tunable photocarrier trapping at the semiconductor/dielectric interface in field-effect transistors (FETs) based on monolayer MoS_2, which is a direct bandgap semiconductor with E_G = 1.84 eV. When light illuminates the phototransistor, it generates photocarriers in the form of electron-hole pairs. If the MoS_2 FET is biased in the ON-state, photocarriers generated in the channel drift towards the respective electrodes under the applied source-to-drain bias, resulting in non-persistent photoconductivity beyond the optical illumination. However, for illuminations in the OFF-state, noticeable shifts are observed in the threshold voltage (V_{TH}) of the device post-illumination, which is ascribed to the photo-gating effect, i.e., trapping of photogenerated carriers at/near the MoS_2/Al_2O_3 interface. Note that in the absence of any gate bias, at equilibrium, the empty trap states are located above the Fermi energy (E_F) while the filled trap states are below E_F. When the phototransistor is illuminated in the ON-state or subthreshold region of device operation, most trap states lie below E_F, making it unlikely for carriers to be trapped. As a result, the photoconductivity is non-persistent beyond optical illumination, and the device returns to its initial state without retaining any optical memory. However, when the phototransistor is illuminated in the OFF-state or depletion region of device operation, most trap states are above E_F, which allows for carrier trapping near the MoS_2/Al_2O_3 interface. The trapping of photo-generated holes is indicated by negative shifts in V_{TH}. With longer illumination, more trap states become occupied, leading to greater shifts in V_{TH}. The process of detrapping can be relatively slow, resulting in persistent photocurrent in the MoS_2 phototransistor. For further details on the photogating effect, additional information can be found in our previous research publications [9-12].

Supplementary Figure 9

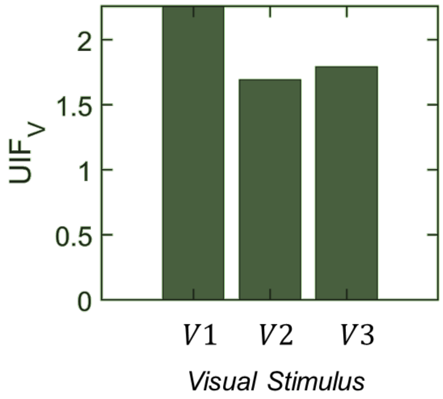

Supplementary Figure 9. Unisensory visual integration. Unisensory integration factor for a given visual stimulus (UIF_V), which is defined as the ratio of the MN's response to dual and single illumination as a function of the strength of that V.

Supplementary Figure 10

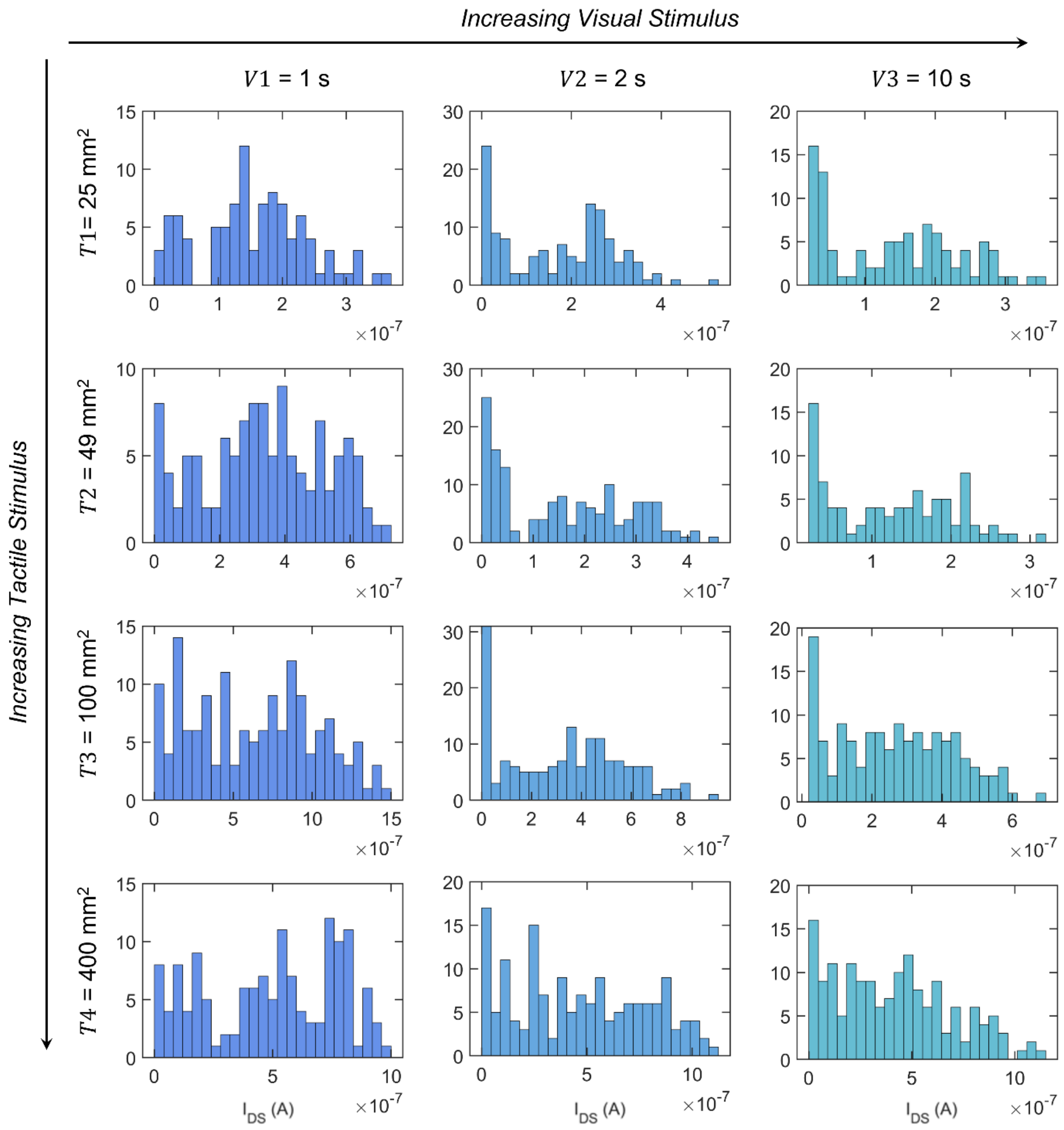

Supplementary Figure 10. Visuotactile response of MN. Histogram of I_{DS} spikes for different combinations of tactile (T) and visual (V) cues.

Supplementary Figure 11

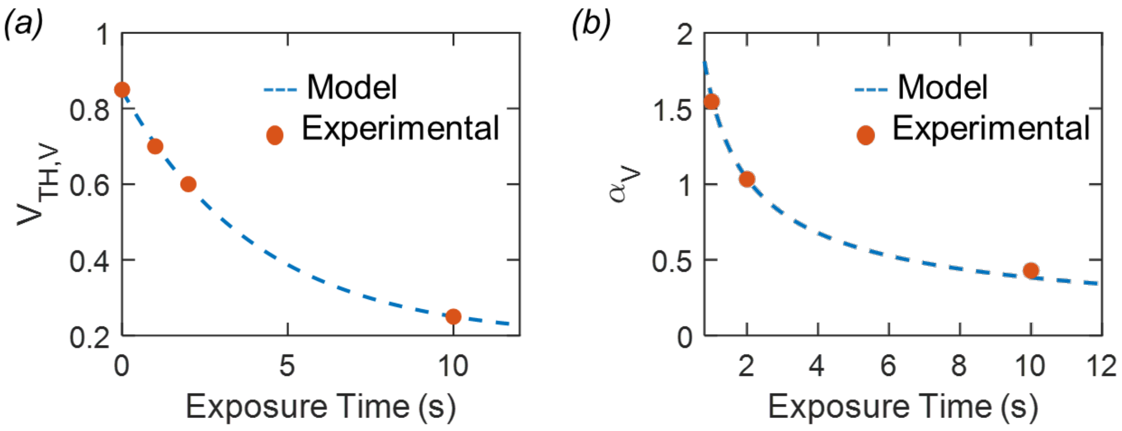

Supplementary Figure 11. Empirical model for visual response. Experimentally obtained and model fitted a) $V_{\mathrm{TH,V}}$ and b) α_{V}.

Supplementary Figure 12

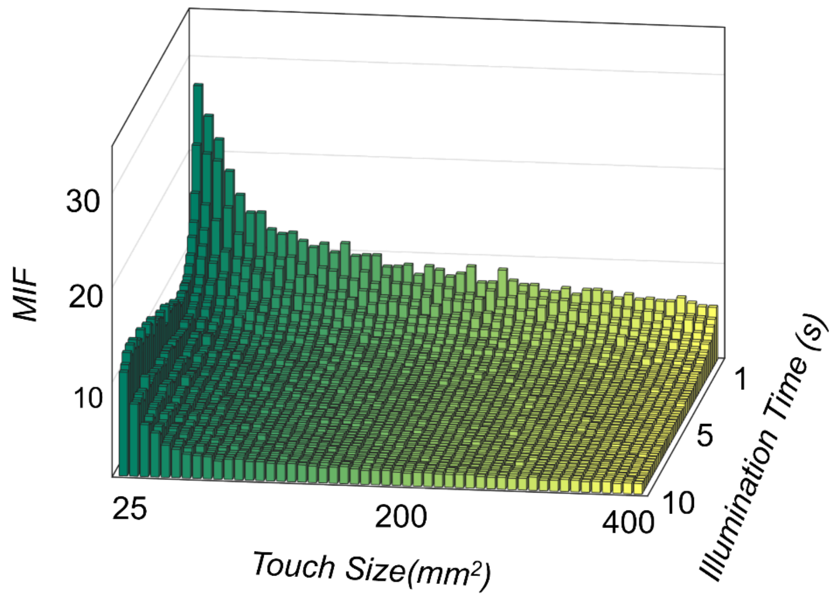

Supplementary Figure 12. Modeling multisensory response. Multisensory integration factor (MIF) obtained from the empirical model for all combinations of visual and tactile stimuli.

Supplementary Figure 13

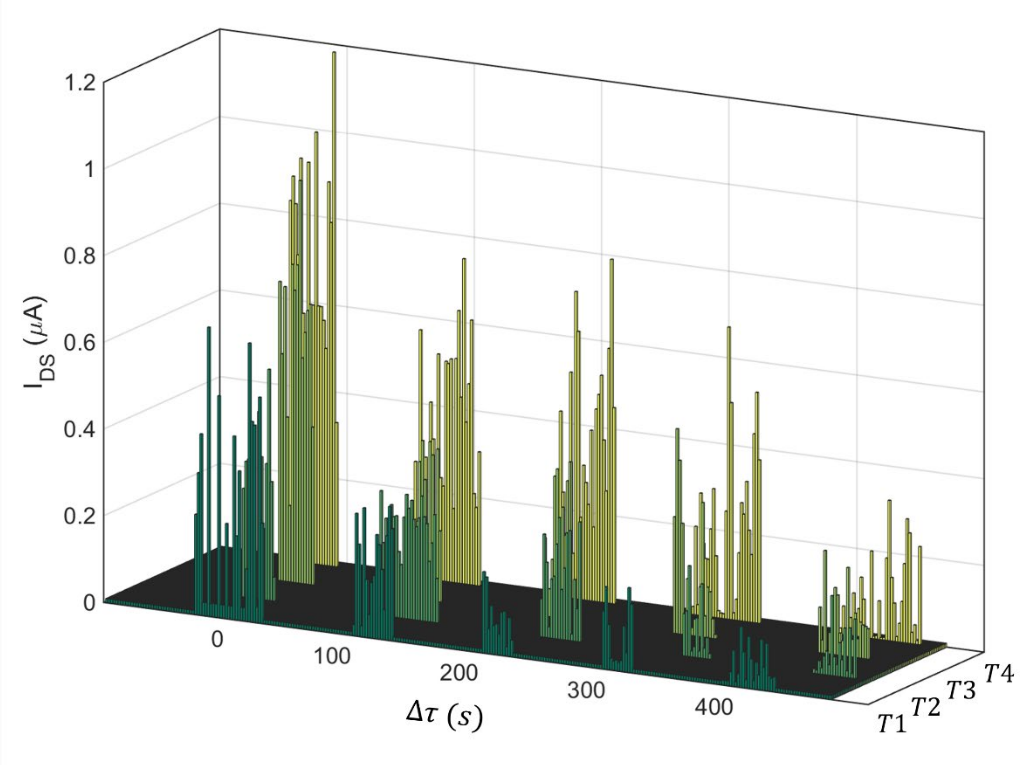

Supplementary Figure 13. Demonstration of temporal congruency by MN. Response of the MN to touch stimuli as a function of lag ($\Delta\tau$) between the tactile (T) and visual (V) stimuli. As the lag increases, the response of the MN shows a monotonic decrease irrespective of the strength of T, which confirms that our artificial MN exhibits temporal congruency. The physical origin of temporal congruency can be attributed to the fact that the persistent photocurrent in MoS_2 photo-memtransistors is a direct consequence of photocarrier trapping at the MoS_2/dielectric interface. With time, the detrapping process gradually resets the device back to its pre-illumination conductance state. This can be regarded as gradual loss of visual memory. Naturally, tactile cues that appear long after the visual cues are expected to evoke significantly reduced responses.

Supplementary Figure 14

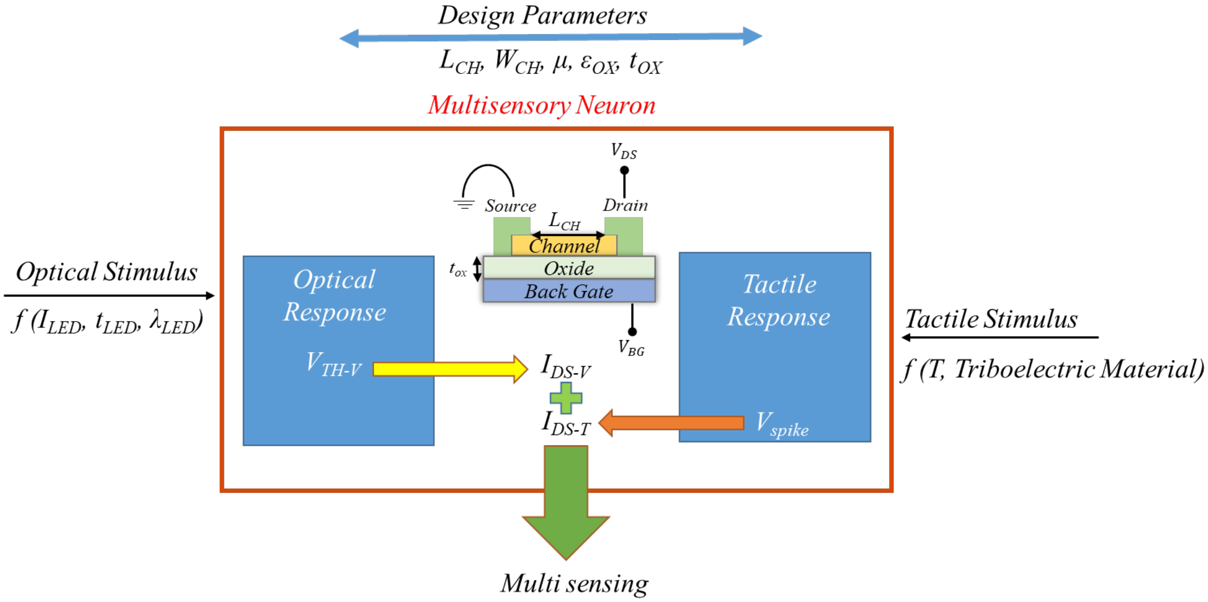

Supplementary Figure 14. Design space for multisensory neuron. Visual response from monolayer MoS_2 photo-memtransistor is obtained due to the photogating effect, which leads to a persistent shift in the threshold voltage ($V_{\mathrm{TH-V}}$) and hence persistent photocurrent ($I_{\mathrm{DS-V}}$). The external variables that influence $V_{\mathrm{TH-V}}$ and $I_{\mathrm{DS-V}}$ are 1) the strength of the optical illumination (I_{LED}), 2) the duration of the optical illumination (t_{LED}), and 3) the wavelength of the optical illumination (λ_{LED}). The biasing condition of the photo-memtransistor can also influence the photo-modulation. Similarly, the tactile response generates an electrical impulse (V_{spike}) to the gate of the MoS_2 photo-memtransistor leading to current spikes ($I_{\mathrm{DS-T}}$) at the output. The external variables that influence V_{spike} and $I_{\mathrm{DS-T}}$ are 1) the surface charge density, which is strongly dependent on the surface contact area (T), and 2) the triboelectric material used (in our case Kapton). As described in the manuscript, V_{spike} follow a random Gaussian distribution with μ_{T} and σ_{T} as the mean and standard deviation, respectively. While the strength of the tactile stimulus (T) is captured through μ_{T}, the uncertainty associated with any triboelectric response is captured through σ_{T}. The dependence of μ_{T} on T is described using an empirical relationship. The fitting parameters, i.e., μ_{01}, T_{01}, μ_{02}, and T_{02}, are expected to show strong dependence on the gate capacitance (C_{G}) since V_{spike} is related to $Q_{\mathrm{tactile}}/C_{\mathrm{G}}$, where Q_{tactile} is the surface charge density that depends on T. Note that C_{G} depends on the thickness and dielectric constant of the gate insulator. Finally, both visual and tactile responses are influenced by the device dimensions, including channel length (L_{CH}), channel width (L_{CH}), and field-effect carrier mobility (μ_{N}). Therefore, to strike a balance between the visual and tactile current response, it is important to design the MoS_2 photo-memtransistor-based multisensory neuron (MN) in such a way that $V_{\mathrm{TH-V}}$ and V_{spike} are of similar magnitudes. To do so, first it is important to have a knowledge of the application environment, i.e., the expected strength of the optical ($I_{\mathrm{LED}}, t_{\mathrm{LED}}, \lambda_{\mathrm{LED}}$) and tactile ($T$) stimuli. Next, using the empirical and physics-based models described in the manuscript, visual and tactile responses can be self-consistently and iteratively solved to arrive at the required device dimensions.

Supplementary Figure 15

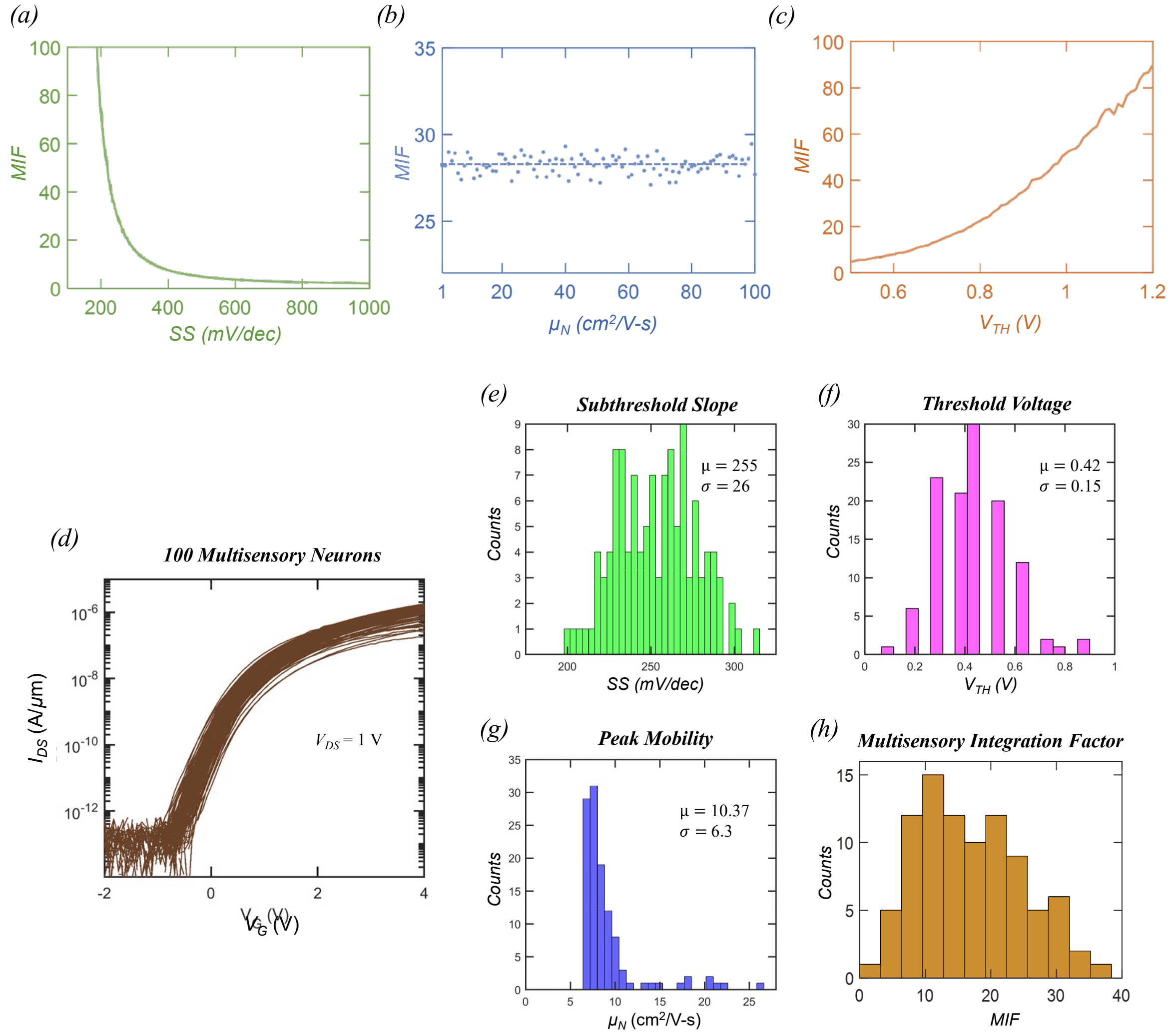

Supplementary Figure 15. Influence of device parameters on multisensory integration. Dependence of MIF on a) SS, b) μ_N, and c) V_{TH} for the weakest tactile and visual stimuli. Clearly, μ_N has the least influence on MIF since it is related to the ON-state performance of the memtransistor, whereas visuotactile responses are generated in the OFF-state of the memtransistor. Therefore, as expected, both V_{TH} and SS have significant impact on MIF. Note that the dependence of MIF on various device related parameters can become a critical design consideration when an ensemble of multisensory neurons is present. d) Transfer characteristics of 100 multisensory neurons. Neuron-to-neuron variation in e) SS, f) V_{TH}, and g) μ_N. h) The projected neuron-to-neuron variation in MIF based on the models discussed in the manuscript.

Supplementary Information 3

Note that the field-effect carrier mobility and subthreshold slope values obtained from the monolayer MoS_2 FETs used in this study may not be the most exceptional values documented in the literature. Nevertheless, they are comparable to the majority of published reports. The champion mobility value for monolayer MoS_2 grown by chemical vapor deposition (CVD) was found to be ~55 $cm^2V^{-1}s^{-1}$ at room temperature with bismuth (Bi) as the contact metal [13]. Another recent study on CVD-grown monolayer MoS_2 with antimony (Sb) as the contact metal reported mobility values of ~50 $cm^2V^{-1}s^{-1}$ [14]. In contrast, the highest mobility value for metal-organic CVD (MOCVD)-grown MoS_2 used in this study was found to be ~ 26 $cm^2V^{-1}s^{-1}$. In an earlier study, we reported a champion mobility value of 46 $cm^2V^{-1}s^{-1}$, which is comparable to the current state-of-the-art values [15]. Note that the mobility of MOCVD-grown monolayer MoS_2 with typical grain sizes of ~1 μm is expected to be lower than CVD-grown single-crystal monolayer MoS_2. However, MOCVD is considered a preferable synthesis technique for manufacturing purposes due to its ability to produce conformal films as opposed to the large triangular flakes typically obtained using CVD [16-18]. Nevertheless, since our MoS_2-based multisensory neuron (MN) is mostly operated in the subthreshold regime, mobility plays a less significant role in multisensory integration factor (MIF), as shown in Supplementary Fig. 15b.

The subthreshold slope (SS) values also differ from the ideal value of 60 $mVdec^{-1}$, which can be explained using the following equation.

$$\mathrm{SS} = \frac{mk_\mathrm{B}T}{q}\ln(10);\ m = \left(1 + \frac{C_\mathrm{S}}{C_\mathrm{OX}} + \frac{C_\mathrm{IT}}{C_\mathrm{OX}}\right);\ C_\mathrm{IT} = qD_\mathrm{IT} \qquad (S1)$$

Here, k_B is the Boltzmann constant, T is the temperature, q is the electron charge, m is the body factor, C_S is the semiconductor capacitance, C_OX is the oxide capacitance, C_IT is the interface trap capacitance, and D_IT is the interface trap density. For ultra-thin body (UTB) semiconductors such as monolayer MoS_2, $C_\mathrm{S} = 0$, and for a clean semiconductor interface, $C_\mathrm{IT} \ll C_\mathrm{OX}$, which ensures that $m = 1$ and $SS = 60$ $mVdec^{-1}$. In this work, the reported median SS value is 255 $mVdec^{-1}$, indicating that C_IT is finite; using Eq. S1, we can extract the median value for D_IT to be ~ 4.5×10^{12} $eV^{-1}cm^{-2}$. In comparison, the D_IT value for the state-of-the-art UTB Si FETs is found to be ~1.5×10^{12} $eV^{-1}cm^{-2}$, leading to an SS of 80 $mVdec^{-1}$ [19] for an effective oxide thickness (EOT) of 4 nm. Therefore, our reported median D_IT value for the monolayer MoS_2 FET is comparable to the

state-of-the art Si FETs. It is possible to achieve better SS by reducing the EOT, which in our case is 20 nm. As mentioned earlier, the MN operates in the off-state; therefore, there will be a significant effect of SS on the multisensory integration factor (MIF). The dependence of MIF on SS can be found in Supplementary Fig. 15a. To achieve MIF > 10, SS values should stay below 400 $mVdec^{-1}$. Interestingly, most of the MoS_2-based MNs exhibited SS values less than 350 $mVdec^{-1}$.

Supplementary Figure 16

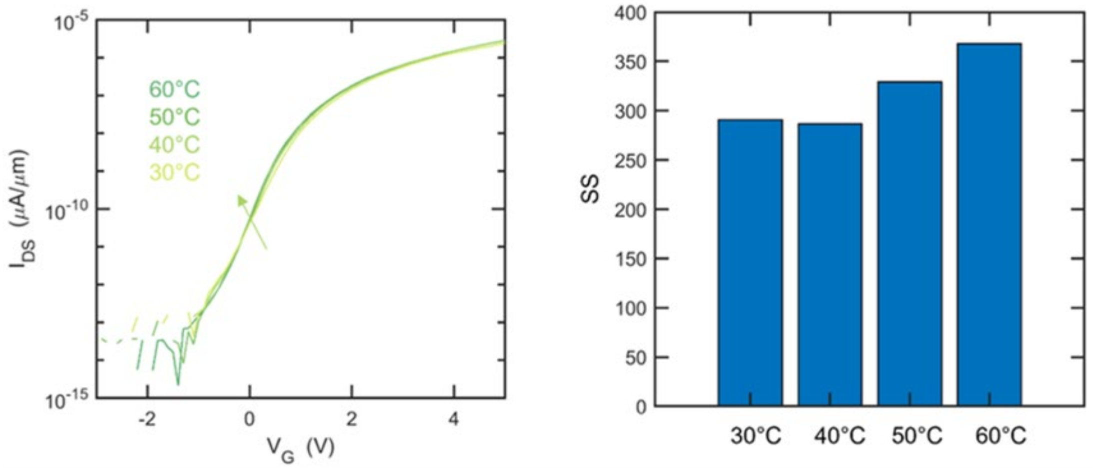

Supplementary Figure 16. Impact of temperature on MN. a) Transfer characteristics of a representative monolayer MoS_2 FET measured at different temperatures, ranging from 30°C to 60°C in steps of 10°C. b) Extracted SS as a function of temperature. The SS value was found to change by only 27%. The temperature dependence of SS will directly influence the MIF. Therefore, depending on the targeted application, it may be necessary to employ standard techniques to mitigate temperature-related drift. This can involve using temperature sensors to measure the ambient temperature and adjusting the circuit parameters or calibration values accordingly. Alternatively, circuit design techniques such as negative feedback, voltage references, and compensation networks can be used to mitigate temperature-related drift. These techniques help stabilize the circuit's performance despite temperature variations. Yet another way is to incorporate guard rings and shielding techniques to minimize the impact of temperature. By using one or more of these techniques, it is possible to mitigate the impact of temperature-related drift on MN.

Supplementary Figure 17

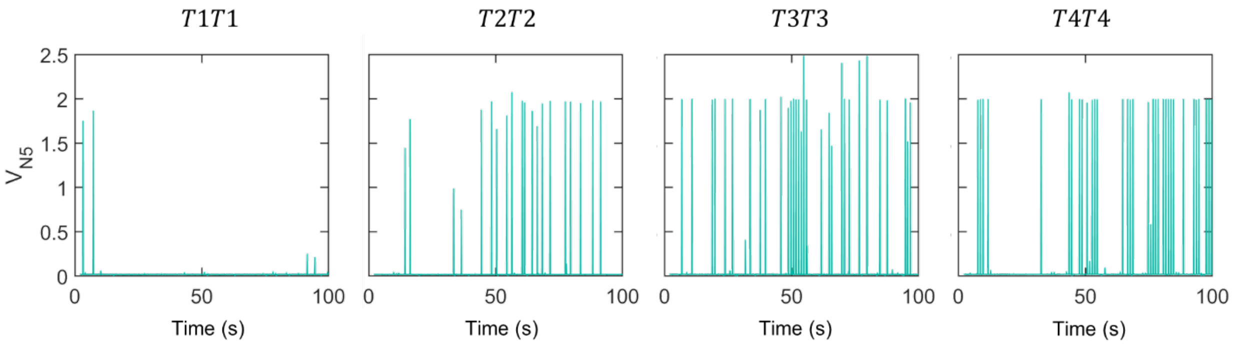

Supplementary Figure 17. Spike encoding for dual touch. Spiking responses of the multisensory neural circuit for dual tactile stimuli inputs starting from $T1T1$ to $T4T4$ in dark.

References

[1] J. You *et al.*, "Simulating tactile and visual multisensory behaviour in humans based on an MoS2 field effect transistor," *Nano Research,* 2023/03/06 2023, doi: 10.1007/s12274-023-5467-7.

[2] C. Jiang *et al.*, "Mammalian-brain-inspired neuromorphic motion-cognition nerve achieves cross-modal perceptual enhancement," *Nature Communications,* vol. 14, no. 1, p. 1344, 2023/03/11 2023, doi: 10.1038/s41467-023-36935-w.

[3] J.-K. Han, S.-Y. Yun, J.-M. Yu, S.-B. Jeon, and Y.-K. Choi, "Artificial Multisensory Neuron with a Single Transistor for Multimodal Perception through Hybrid Visual and Thermal Sensing," *ACS Applied Materials & Interfaces,* vol. 15, no. 4, pp. 5449-5455, 2023/02/01 2023, doi: 10.1021/acsami.2c19208.

[4] J. Yu *et al.*, "Bioinspired mechano-photonic artificial synapse based on graphene/MoS₂ heterostructure," *Science Advances,* vol. 7, no. 12, p. eabd9117, 2021, doi: doi:10.1126/sciadv.abd9117.

[5] G. Chen *et al.*, "Temperature-controlled multisensory neuromorphic devices for artificial visual dynamic capture enhancement," *Nano Research,* vol. 16, no. 5, pp. 7661-7670, 2023/05/01 2023, doi: 10.1007/s12274-023-5456-x.

[6] L. Sun, Y. Du, H. Yu, H. Wei, W. Xu, and W. Xu, "An Artificial Reflex Arc That Perceives Afferent Visual and Tactile Information and Controls Efferent Muscular Actions," *Research,* vol. 2022, 2022, doi: doi:10.34133/2022/9851843.

[7] L. Liu *et al.*, "Stretchable Neuromorphic Transistor That Combines Multisensing and Information Processing for Epidermal Gesture Recognition," *ACS Nano,* vol. 16, no. 2, pp. 2282-2291, 2022/02/22 2022, doi: 10.1021/acsnano.1c08482.

[8] M. Wang *et al.*, "Gesture recognition using a bioinspired learning architecture that integrates visual data with somatosensory data from stretchable sensors," *Nature Electronics,* vol. 3, no. 9, pp. 563-570, 2020/09/01 2020, doi: 10.1038/s41928-020-0422-z.

[9] A. Dodda *et al.*, "Active pixel sensor matrix based on monolayer MoS2 phototransistor array," *Nature Materials,* vol. 21, no. 12, pp. 1379-1387, 2022/12/01 2022, doi: 10.1038/s41563-022-01398-9.

[10] S. Subbulakshmi Radhakrishnan *et al.*, "A Sparse and Spike-Timing-Based Adaptive Photoencoder for Augmenting Machine Vision for Spiking Neural Networks," *Advanced Materials,* vol. 34, no. 48, p. 2202535, 2022, doi: https://doi.org/10.1002/adma.202202535.

[11] S. Subbulakshmi Radhakrishnan, A. Dodda, and S. Das, "An All-in-One Bioinspired Neural Network," *ACS Nano,* vol. 16, no. 12, pp. 20100-20115, 2022/12/27 2022, doi: 10.1021/acsnano.2c02172.

[12] A. Dodda *et al.*, "Bioinspired and Low-Power 2D Machine Vision with Adaptive Machine Learning and Forgetting," *ACS Nano,* vol. 16, no. 12, pp. 20010-20020, 2022/12/27 2022, doi: 10.1021/acsnano.2c02906.

[13] P.-C. Shen *et al.*, "Ultralow contact resistance between semimetal and monolayer semiconductors," *Nature,* vol. 593, no. 7858, pp. 211-217, 2021/05/01 2021, doi: 10.1038/s41586-021-03472-9.

[14] W. Li *et al.*, "Approaching the quantum limit in two-dimensional semiconductor contacts," *Nature,* vol. 613, no. 7943, pp. 274-279, 2023/01/01 2023, doi: 10.1038/s41586-022-05431-4.

[15] A. Sebastian, R. Pendurthi, T. H. Choudhury, J. M. Redwing, and S. Das, "Benchmarking monolayer MoS2 and WS2 field-effect transistors," *Nature Communications,* vol. 12, no. 1, p. 693, 2021/01/29 2021, doi: 10.1038/s41467-020-20732-w.

[16] L. Yu *et al.*, "Design, Modeling, and Fabrication of Chemical Vapor Deposition Grown MoS2 Circuits with E-Mode FETs for Large-Area Electronics," *Nano Letters,* vol. 16, no. 10, pp. 6349-6356, 2016/10/12 2016, doi: 10.1021/acs.nanolett.6b02739.
[17] K. Kang *et al.*, "High-mobility three-atom-thick semiconducting films with wafer-scale homogeneity," *Nature,* vol. 520, no. 7549, pp. 656-660, 2015/04/01 2015, doi: 10.1038/nature14417.
[18] K. K. H. Smithe, S. V. Suryavanshi, M. Muñoz Rojo, A. D. Tedjarati, and E. Pop, "Low Variability in Synthetic Monolayer MoS2 Devices," *ACS Nano,* vol. 11, no. 8, pp. 8456-8463, 2017/08/22 2017, doi: 10.1021/acsnano.7b04100.
[19] C. Min, T. Kamins, P. V. Voorde, C. Diaz, and W. Greene, "0.18-μm fully-depleted silicon-on-insulator MOSFET's," *IEEE Electron Device Letters,* vol. 18, no. 6, pp. 251-253, 1997, doi: 10.1109/55.585344.
