# Supplementary material for: A bio-inspired visuotactile neuron for multisensory integration
Source: Nat Commun. 2023 Sep 15;14:5729. doi: 10.1038/s41467-023-40686-z (PMC10504285; doi:10.1038/s41467-023-40686-z)
Supplement: Supplementary file 3 — Description of Additional Supplementary Files [file 41467_2023_40686_MOESM3_ESM.docx]

File Name: Supplementary Video 1

Description: Demonstration of single touch input ($T$) on the triboelectric tactile sensor.

File Name: Supplementary Video 2

Description: Demonstration of dual touch input ($TT$) on the triboelectric tactile sensor.
